# ILDgenDB: integrated genetic knowledge resource for interstitial lung diseases (ILDs)

**DOI:** 10.1093/database/bay053

**Published:** 2018-06-09

**Authors:** Smriti Mishra, Mohammad I Shah, Malay Sarkar, Nimisha Asati, Chittaranjan Rout

**Affiliations:** 1 Department of Biotechnology and Bioinformatics, Jaypee University of Information Technology, Waknaghat, Solan, Himachal Pradesh 173234, India; 2 Department of Pulmonary Medicine, Indira Gandhi Medical College, Shimla, Himachal Pradesh 171001, India

## Abstract

Interstitial lung diseases (ILDs) are a diverse group of ∼200 acute and chronic pulmonary disorders that are characterized by variable amounts of inflammation, fibrosis and architectural distortion with substantial morbidity and mortality. Inaccurate and delayed diagnoses increase the risk, especially in developing countries. Studies have indicated the significant roles of genetic elements in ILDs pathogenesis. Therefore, the first genetic knowledge resource, ILDgenDB, has been developed with an objective to provide ILDs genetic data and their integrated analyses for the better understanding of disease pathogenesis and identification of diagnostics-based biomarkers. This resource contains literature-curated disease candidate genes (DCGs) enriched with various regulatory elements that have been generated using an integrated bioinformatics workflow of databases searches, literature-mining and DCGs–microRNA (miRNAs)–single nucleotide polymorphisms (SNPs) association analyses. To provide statistical significance to disease-gene association, ILD-specificity index and hypergeomatric test scores were also incorporated. Association analyses of miRNAs, SNPs and pathways responsible for the pathogenesis of different sub-classes of ILDs were also incorporated. Manually verified 299 DCGs and their significant associations with 1932 SNPs, 2966 miRNAs and 9170 miR-polymorphisms were also provided. Furthermore, 216 literature-mined and proposed biomarkers were identified. The ILDgenDB resource provides user-friendly browsing and extensive query-based information retrieval systems. Additionally, this resource also facilitates graphical view of predicted DCGs–SNPs/miRNAs and literature associated DCGs–ILDs interactions for each ILD to facilitate efficient data interpretation. Outcomes of analyses suggested the significant involvement of immune system and defense mechanisms in ILDs pathogenesis. This resource may potentially facilitate genetic-based disease monitoring and diagnosis.

Database URL: http://14.139.240.55/ildgendb/index.php

## Introduction

Interstitial lung diseases (ILDs), also known as diffuse parenchymal lung diseases, encompass a diverse group of acute and chronic lung disorders with known and unknown causes that mainly involve interstitium of the lungs (1). Occasionally, these diseases also involve the alveoli, peripheral airways, vessels and pleura. According to the study on global burden of disease (1990–2013), these diseases are one of the ten causes of increased global deaths (2). ILDs cases are very frequent in developing countries due to the use of tobacco, cooking by coal and exposure to environmental hazards (3). Incidence and mortality rates are found to be increased with age, exposure and other similar risk factors. Though abundance data on these diseases has not been available in India, studies from 1979 to 2016 indicated that the majority of ILDs patients suffered from idiopathic pulmonary fibrosis (IPF) and hypersensitivity pneumonitis (HP) (4).

The diagnosis of ILDs in developing countries is based on elicitation of thorough and comprehensive clinical history, including evidence of occupational and environmental exposures, drug intake, familial history, multi-systemic examination, chest X-ray (CXR) and high-resolution computed tomography (HRCT). Multi-disciplinary discussion among pulmonologists, radiologists and pathologists is essential for the accurate diagnosis of these diseases (5), but it is generally not in practice in developing countries. Under-diagnosis and misdiagnosis are common incidences leading to delay in effective treatment. Over-dependency on CXRs for the diagnosis is considered to be an important factor for misdiagnosis as this technique can only predict 10% of ILDs cases (6). Moreover, ILDs are often misdiagnosed as tuberculosis (TB) in TB-endemic countries (7). Unavailability of specialized tests such as HRCT scanning, spirometry, diffusion capacity, bronchoscopy, surgical lung biopsy and video-assisted lung biopsy are the other limitations toward an early and accurate diagnosis. There is also a scarcity of expert pathologists who can interpret surgical lung biopsy specimens accurately (7). Furthermore, in elderly patients, lung biopsy is often not possible due to the presence of frequent co-morbidities. Variability in the clinical courses is another important fact about these diseases. A subset of patients often shows a rapid progression of the disease whereas it may be slow for the others (8). Therefore, biomarker-based ILDs management is expected to solve most of these problems by making an early and accurate diagnosis, identifying the rapid progressing phenotypes and monitoring the prognosis of disease so that the treatment may be carried out in an efficient manner (9).

Studies have shown the evidence of genetic inheritances in familial silicosis, cystic fibrosis, bronchitis (10, 11) and IPF (12). Genetic studies performed on animal model have given clear insights into the involvement of a few genes on pathogenesis, prognosis and etiology of the ILDs (13). Therefore, a comprehensive and comparative analysis of genes and other associated genetic data have potential to provide candidate molecular diagnostic biomarkers. Keeping in view of the substantial utility of genetic data, a highly curated comprehensive knowledge resource ILDgenDB has been developed. The major applications of this web resource have also been provided. This resource provides literature-curated 299 genes, their annotations, associated genetic data and involvement of these genetic elements in ILDs pathogenesis. Functional annotation of these genes was performed using gene ontology (GO) and pathways mapping, and association of these genes with single nucleotide polymorphism (SNP) and microRNAs (miRNAs) were also analyzed. Biomarkers and information on their association with ILDs diagnosis were provided. All these data were incorporated to the developed knowledge resource. Hyperlinks to other well-known servers such as NCBI, UniProt, Ensembl, dbSNP, PDB, KEGG and OMIM are also provided to facilitate advanced searching in ILDgenDB knowledge resource. Protocols for the identification of potential biomarkers in ILDs monitoring and management have also been proposed. This manually curated knowledge resource would promote important traits to understand ILDs pathogenesis. All the datasets including analyzed results and biomarkers are freely accessible through extensive query and browsing options.

## Materials and methods

### Data collection and integration

#### Disease candidate genes identification

**Figure 1.**
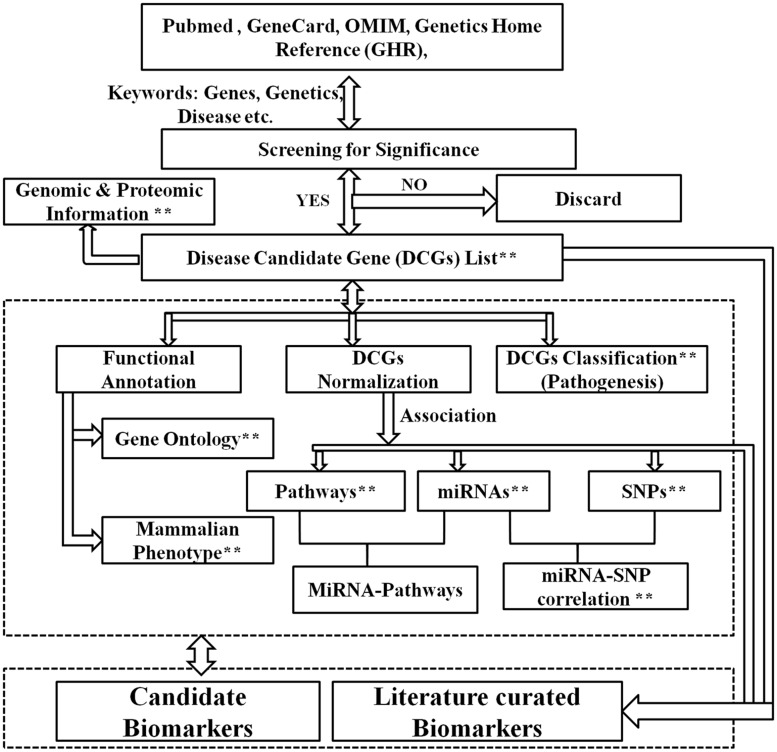
Architecture of ILDgenDB knowledge resource. **The query terms that can be used to search the knowledge resource: name of each class of data such as gene, disease, category, biomarker, MiRNA AND/OR SNP, GO and phenotype may be used as query.

Schematic representations of different types of data incorporated in the knowledge resource are given in Figure 1. A systemic review of literature using exhaustive query was conducted in PubMed to find out initially the genes involved in ILDs. Genes associated with ILDs were manually curated and validated through published literature. These validated genes were enriched through information available in the gene-disease association databases such as KEGG, GAD, OMIM, GHR, CTD, DISEASE and GeneCards (Supplementary Table S1). The final list of validated genes associated with ILDs were considered as disease candidate genes (DCGs) and incorporated into ILDgenDB. Genes without direct association with the disease, or having ambiguity or redundant information were excluded. Based on the involvement in disease pathogenesis, these DCGs were grouped into five different categories namely, mutation, differential expression, biomarker and genetic testing, therapeutic targets and others. Genomic and proteomic information of these DCGs were mined from standard resources including NCBI, UniProt, PDB, Ensemble etc. (Supplementary Table S1). A sample advanced query used to identify genes involved in ILDs is given as follows:

‘Interstitial lung disease’[All Fields] AND (‘genes’[MeSH Terms] OR ‘gene’[All Fields]) AND ‘genetics’[All Fields] OR ‘genetics’[MeSH Terms])

#### ILDs specificity index calculation of DCGs

There are a few DCGs associated with more than one ILD’s subtype (e.g. SFTPC gene is involved in many different ILDs such as lymphocytic interstitial pneumonia, nonspecific interstitial pneumonia, children's ILD (ChILD), pulmonary surfactant dysfunction and IPF. ILDs specificity index (ILDsi), adopted from DisGeNET (Supplementary Table S1), of each DCG was calculated to determine the extent of DCGs-disease association [equation (1)].

(1) $$\text{ILDsi}=\left(\frac{\;{\text{log}}_{2}(\text{Ng}/\text{Nd})}{\;{\text{log}}_{2}(1/\text{Nd})}\right),$$

where Ng is the number of diseases associated with a gene and Nd is the total number of diseases in ILDgenDB.

The ILDsi score ranges from 0 to 1, and the lower is the score, the greater number of diseases is found to associate with the DCG.

#### DCG enrichment using hypergeometric test

To determine extent of a DCG studied for a group of ILDs, a hypergeometric test has been performed (14). This test results a scores (*P*-value) range from 0 to 1, for each gene-disease association pair and takes into account the number of publications supporting the association. This test statistically determines that the association between an ILD and DCG is significantly higher than by random chance. The lower is the *P*-value; the more significant is the association.

#### Gene normalization, and genomic and proteomic annotation

**Figure 2.**
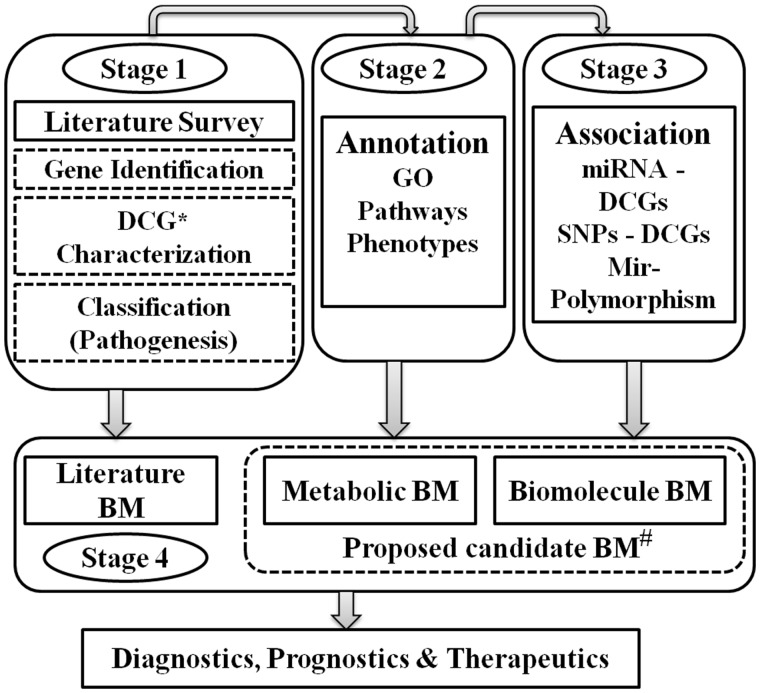
Stage-wise workflow of ILDgenDB knowledge resource. *DCG: disease candidate gene; #BM: biomarkers: analysis of genetic data for the prediction of potential biomarkers.

For the standardized recognition and cross-referencing of each DCGs, gene aliases, currently approved symbol, previous symbols and the standard IDs mentioned in different databases such as Entrez, InterPro, Ensembl, HGNC, UniProt, OMIM, VEGA and MGI were included (Figure 1). For all the DCGs, important information such as functions, cryptogenic location, nucleotide and amino acid sequences, pathways, mammalian phenotypes, mRNAs, domains, protein family, pathogenesis and other relevant information were also incorporated (Figure 2). Current *Homo sapiens* Release 108, GRCh38.p7 was used for annotation.

#### GO analysis and association of miRNA and SNP with DCGs

To characterize the functional importance of DCGs, the GO analysis was performed using AmiGO, Ensembl BioMart, DAVID and PANTHER (Supplementary Table S1). Only significant GO terms with a cutoff *P*-value of ≤0.05 were considered, and the redundant data were removed. Although cutoff *P*-values 0.01 and 0.07 were also evaluated, the GO analysis with *P*-value ≤0.05 provided optimal results. The DCGs involvements with the biological processes, molecular functions and cellular component categories were added to the knowledge resources after verifying their associations with diseases.

Prediction of miRNAs interacting with mRNA (each of validated 299 DCGs) was performed using miRNA prediction tools and databases such as PITA, miRwalk, mirBase, starBase and targetScan (Supplementary Table S1). miRNAs were scanned for the evidences of experimental verification and clinical significance with reference to the miRNA-mRNA (DCGs) association. The identified potential miRNA list was compared with mirBase for subsequent validation (Figure 1). After removal of ambiguous and duplicate values, genomic coordinates and mature sequences of the miRNAs were included in ILDgenDB.

Identification of SNP-DCGs association was performed using dbSNP. Only clinically significant SNPs and their annotations were used for the filtrations of potential SNPs (Supplementary Table S1). The association among miRNA-SNPs-DCGs was also mined using the miRdSNP database (Supplementary Table S1). Clinical significance and genomic location of SNPs were analyzed, and insignificant SNPs were excluded. Only unique and clinically significant miRNA-SNPs-DCGs associations were incorporated into ILDgenDB.

#### Biomarkers

The PubMed was consecutively mined using a specific medical subject heading (MeSH) terms representing different ILDs and their established biomarkers. Different keywords (biomarkers, genetic testing, disease diagnostics, molecular diagnostics etc.) were used separately for retrieving data on reported biomarkers. An example of standard query is given as follows:

‘Interstitial lung disease’[All Fields] AND ‘biomarker’[All Fields]

These biomarkers were grouped according to their cellular sources and biological sample (e.g. plasma, serum, biopsy samples etc.). The biomarkers having roles in pathogenesis were further subcategorized on the basis of their annotation available in the literature as gene, protein and/or disease specific (Figure 2). The remarks and references related to each biomarker were also incorporated in ILD resource for better understanding of their roles and applications.

### Data access

**Figure 3.**
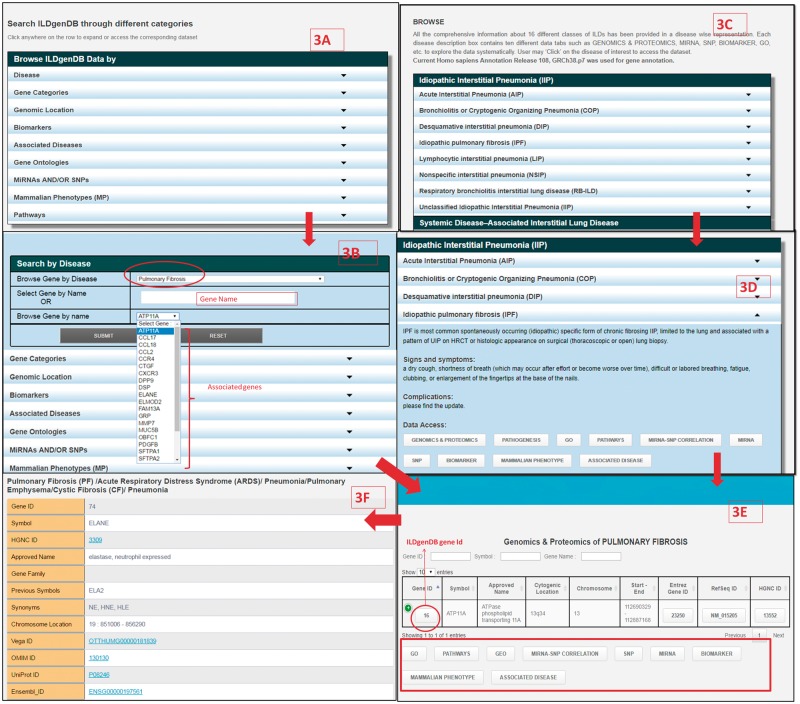
Retrieval of information from ILDgenDB knowledge resource using ‘Search’ and ‘Browse’ options; (**A**) exploring different categories of data using ‘Search’ option; (**B**) determining gene’s association with disease by choosing ‘Disease’ option in ‘A’ with ‘IPF’ as query term and selecting gene name as ‘ATP11A’; (**C**) exploring each disease classes information of ILDgenDB using “Browse” option; (**D**) accessing 10 different categories of data related to a disease ‘IPF’ by exploring hyperlinked each tab; (**E**) output of genomic and proteomics tab and similar output is also obtained from (B); and (F) output page displaying all related information to a gene (ELANE) after clicking on its ‘Gene ID’.

To facilitate efficient access and retrieval of data, the ILDgenDB interface provides two advanced features, i.e. ‘Browse’ and ‘Search’ (Figure 3). More detailed information on these options is available in ILDgenDB tutorials accessible at http://14.139.240.55/ildgendb/tutorial.html. Integrated information of the knowledge resource can be browsed by disease name directly or using query terms with AND/OR options (Figures 1 and 3). Different categories of data are interlinked so that the user can easily navigate to explore all search options.

### Web implementation

The architecture of ILDgenDB knowledge resource is provided in Figure 1, which was implemented on MySQL server to provide fast executions of multiple queries defined by users. The first/user level was supported by JavaScript, PHP, HTML and cascading style sheets. This knowledge resource comprised of two different layers: data and application levels. The former stores basic genomic and proteomic information of the DCGs, while the later contains all the analysis outcomes performed on DCGs. These data are presented in an interactive manner to facilitate easy access and analysis.

#### Disease gene network viewer

The disease-gene network viewer, Network Visualization (NetViz), is a JavaScript-based application that is created to provide a visual display of gene-disease association network for a given disease (http://14.139.240.55/ildgendb/netviz.php). NetViz provides two options for the data visualization. In the first option, association among ILDs, its DCGs and SNPs/miRNAs are provided, while literature curated ILD-DCGs association based on hypergeometric *P*-value are provided in the second option.

## Results and discussion

Disease-specific genetic data resources containing genes, pathways, SNPs, mRNAs, miRNAs etc. information assist in the identification of biomarkers, therapeutics targets and other molecular networks (15). Several such data resources have been assisting in better disease monitoring and diagnosis (16). However, the majority of resources containing these types of data are related to cancers or diseases caused by bacteria and viruses (17). ILDgenDB is the first knowledge resource containing reported DCGs, SNPs, miRNAs, biomarkers, different pathways and other relevant data related to different types of ILDs (Figure 2, Stage 1). A literature survey was performed in PubMed and PMC to identify DCGs involved in ILDs. Genetic data and their role in disease pathogenesis were also deciphered. The sources or references about the DCG-disease association are also provided for every gene in the ILDgenDB resource.

Annotation and characterization of all DCGs were carried out by performing GO term analysis, phenotype analyses and pathways mapping (Figure 2, Stage 2). Association of DCGs with miRNAs and SNPs was analyzed and incorporated to ILDgenDB after verification (Figure 2, Stage 3). Biomarkers data collected from the literature was incorporated into the resource. Novel disease-specific potential biomarkers determined through analyses and integration of ILDs genetic data were also proposed (Figure 2, Stage 4). The major applications of this knowledge resource are presented below.

### ILDgenDB web application

#### ‘Search’ utility

ILDgenDB provides an extended interface to explore its data through ten diverse query terms, e.g. disease name, gene name, miRNA and/or SNP etc. (Figures 1 and 3A). For example, a user can select disease name ‘IPF’ from the drop-down list of ‘Browse Gene by Disease’ option, then the name of all associated genes will be automatically displayed in the next drop down list. Any gene symbol, e.g. ‘ATP11A’ may directly be entered (Figure 3B). Associated reference annotation of the gene such as ontology, phenotype, pathway information, miRNA and SNP information are cross-linked with results (Figure 3E). Output page (Figure 3F) provides detailed information about selected gene by clicking on ‘Gene ID’ (Figure 3E). Detailed information of DCGs such as mRNA, CDS, protein sequence, genomic location and some useful external links to other resources are also provided (Figure 3F). Details of all the options in ‘Search’ utility to extract optimal information are provided in the tutorial of ILDgenDB. Genetic data related to individual ILD may also be retrieved through ‘Browse’ utility using the name of diseases such as acute interstitial pneumonia, IPF etc. (Figure 3C).

#### Disease gene network viewer (NetViz)

**Figure 4.**
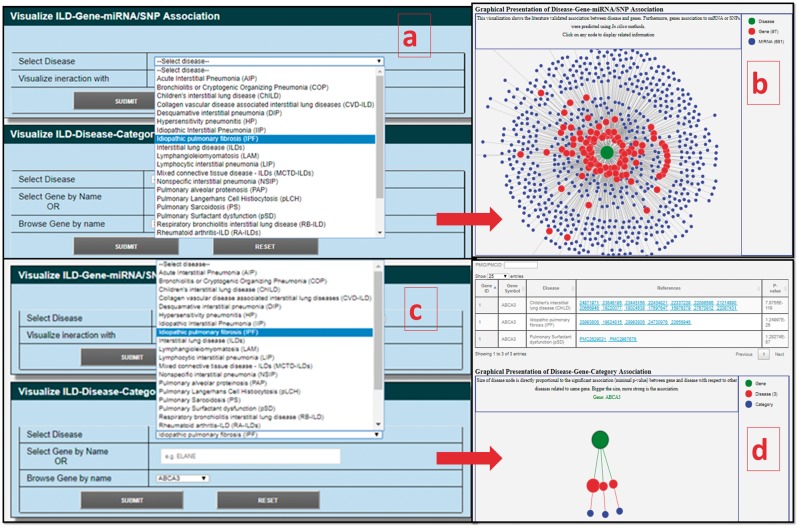
Interactive NetViz of DCGs–miRNAs/SNPs and DCGs–ILDs association based on published articles; (**A**) query selection page for disease as ‘IPF’ and interaction network as ‘miRNA’; (**B**) result page for DCGs–miRNAs association for selected query term; (**C**) query selection box for disease as ‘IPF’ and DCG as ‘ABCA3’ for ILD-disease-category literature association; (**D**) result page for selected query, a table shows all the references associated with the DCGs with queried and other ILDs with *P*-values, and graphical representation of DCG-ILD association.

NetViz is an interactive visualization for different ILD subtypes. In the first option (ILD-Gene-miRNA/SNP Association), user can select ILD-type of interest from drop down menu and then select to view network either by opting ‘miRNA’ or ‘SNP’ (Figure 4A). Result page redirects user to the interaction graph and assists to identify SNP or miRNA association with one or many DCGs. The interaction graph is a force directed plot and smaller length of edges among internodes shows the higher number of miRNA/SNPs interactions that DCGs share with the ILDs (Figure 4B). These associations could be further studied for their regulatory role in pathogenesis or as biomarkers. In the second option (ILD-Disease-Category Literature Association), user can select ILDs type and DCG either by dropdown menu or by entering it manually (Figure 4C). Result page will provide a table for the selected DCG and associated PubMed ids, and hypergeometric *P*-values with respect to different ILDs. The graphical representation of disease-gene-category association will be shown after the table. In the graph, size of disease node is directly proportional to the significant association (minimal *P*-value) between gene and disease with respect to other diseases related to same gene (Figure 4D).

### DCGs categorization and functional annotation, and their association with regulatory elements

On the basis of involvement in ILDs pathogenesis, the DCGs were grouped into five categories such as therapeutic targets, biomarker and genetic testing, differential expression, mutations and others. On the basis of their associated pathways and interaction with other regulatory elements, DCGs including IL-10, transforming growth factor β (TGF-β), tumor necrosis factor (TNF) etc. are grouped as potential therapeutic targets. Many DCGs that were showing up or down regulation in diseased versus control are grouped as differential expression. BDNF and many other genes expression profiles were already validated for their role in pulmonary diseases (18). Few established DCGs such as surfactant proteins (SFTPB, SFTPC), ABCA3, TERT and TERC were already verified for their differential functional and structural importance in lung diseases (19–21). The role of mutation in above genes was also verified in smoking-related ILDs (22). These outcomes have shown that experimental validation of these DCGs can be useful for exposure induced ILDs. These verified DCGs may be considered as potential biomarkers for the diagnosis of ILDs or their sub-types. Few promising biomarkers are lung epithelium-derived proteins such as KL-6, surfactant proteins SP-D, SP-A and CCL18 (23). Genetic biomarkers such as SNPs and miRNA may help to detect subtypes of patients with different needs of management and treatment strategies (23). These categories are not mutually exclusive, and DCGs found to have either indirect role or co-expressed with other key genes in ILDs pathogenesis were classified as other (type of association).

#### Functional annotation of DCGs with GO analysis

Functional and enrichment analyses of DCGs were performed to discover an association between DCGs and corresponding gene product's biology. The biological processes, molecular functions and cellular components of all the DCGs were analyzed to identify the significant representation of GO terms. Outcomes of biological processes have shown that the maximum number of DCGs such as IL-4, IL-7, IL-8, IL-10 and CCL-ligands are mapped with defense response (90), response to external stimulus (84) and immune system process (74) (Supplementary Figure S1C). Identified cellular components for the majority of DCGs are extracellular region part (148), extracellular space (112) and few are from the intrinsic component of the membrane (24) (Supplementary Figure S1B). Results of molecular function have shown that the most of DCGs are mapped with cytokine activity (32), small molecule binding (12), chemokine receptor binding (11) and nucleoside binding (7) (Supplementary Figure S1A). These results suggest the involvement of immune system and molecular binding in ILDs pathogenesis.

#### Pathways mapping of DCGs

ILDs are progressive diseases that are associated with inflammation and lung fibrosis. Targeting the inflammation and fibrotic pathways may assist in disease therapeutics (24, 25). ILDgenDB provides pathways mapping information of DCGs. Analyses have indicated that the DCGs such as TGF-β, SMAD, MMP-2 and MMP-9 are mapped with different pathways related to stimulation, signaling cascades and pro-fibrotic protein expression. The role of these pathways was reported in early and late-onset ILDs pathogenesis (24, 26). Pathways analyses of the DCGs have also suggested their significant involvement in the immune system (134 DCGs), innate immune system (91 DCGs), signal transduction (67 DCGs) and cytokine signaling (59 DCGs). Chemokine and cytokine signaling are the other potential pathways where few important DCGs such as IL9, IL10, TNF and CCL11 are involved. These outcomes suggest that the cytokine-cytokine receptor interactions may be targeted for disease diagnosis as these are actively participated in adaptive inflammatory host defense, and the development and repair processes which are critical for the progression of ILDs. Targeting selective immune processes may give new insight to the novel therapeutics (Supplementary Table S2).

### Association of DCGs with miRNAs and SNPs

#### miRNAs and DCGs association

Interactions of miRNA(s) with DCGs involved in ILDs provide insights on disease pathogenesis and prognosis (27, 28). To gain a global view of the molecular control networks, miRNA and DCGs association analysis was performed (Figure 4; Supplementary Table S5). The current analyses indicated that hsa-miR-335, hsa-miR-26b, hsa-let-7 and hsa-miR-30 target approximately 65, 34, 33 and 26 distinct DCGs, respectively. Differential expression of these target DCGs was verified in many ILDs such as IPF, systemic sclerosis and acute lung injury (29, 30). Up-regulation in miR-126, miR-145a, miR-21, miR-221/222, miR-106a, miR-155 and down-regulation in let-7, miR-20b and miR-133a were reported in pathogenesis of lung’s inflammatory responses. Similarly, miR-155, miR-29, miR-200, miR-21 and miR-326 were reported to have a very significant role in IPF (31). Down-regulation in Let-7f, miR-30c, miR-22 and up-regulation of miR-451 and miR-322 were found to be associated with pulmonary hypertension which is closely related to ILDs. Pathways analysis of let-7 family miRNAs suggested that they may influence hepatic fibrogenesis through activation of transforming growth factor β (TGF-β) signaling in hepatic satellite cells (32). Six distinct miRNAs (miR-21, miR-16, miR-146a, miR-155, miR-126 and miR-223) were reported as potential biomarkers for different diseases (33) including lung disorders.

**Figure 5.**
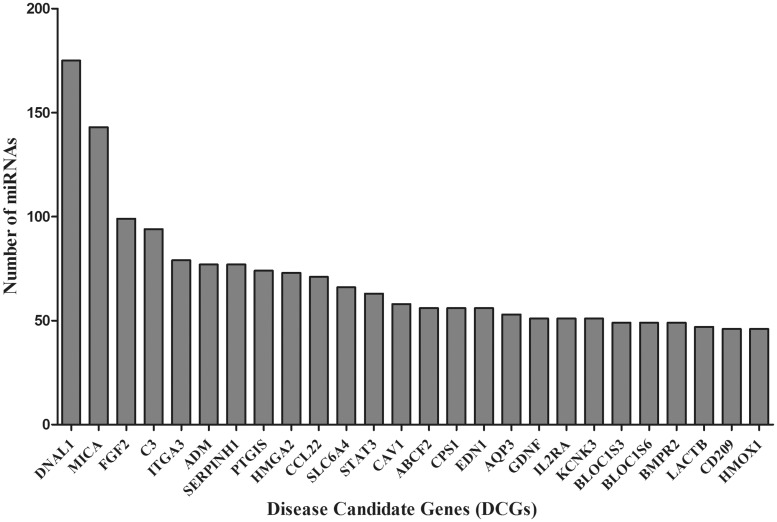
Top ranked DCGs targeted by multiple number of miRNAs.

Furthermore, the DCGs that possibly interact with multiple miRNAs were also analyzed. DNAL1, MICA, FGF2, C3 and ITGA3 are the top five DCGs which are targeted by 175, 143, 99, 94 and 79 miRNAs, respectively (Figure 5). The MICA gene was known to carry miRNA-mediated immune effect in lung tissues (34). Other DCGs including HMOX1, STAT3 etc. were proved to have significant roles in the pathogenesis of IPF (35), immune deficiency disorder and autoimmune ILDs (36). Constitutive expressions of TGF-β1, IL6 and other interleukin families were experimentally verified (37, 38). Our findings may serve as a starting point to the researchers working on the genetics of ILDs. The proposed new target may act as potential biomarker to improve disease management and diagnostics. The regulatory role of miRNAs (hsa-miR-335, hsa-miR-26b, hsa-let-7 and hsa-miR-30 etc.) and altered biological pathways of top-ranked DCGs (DNAL1, MICA, FGF2, C3, ITGA3 etc.) could be explored as potential markers for ILDs diagnosis (Figure 5).

#### SNP and DCGs association

SNPs associated with DCGs were identified using dbSNP and Ensemble databases. These SNP-DCGs associations were included into the ILDgenDB resource with their clinical significance. Genetic studies substantiated that SNP-gene associations could play a significant role in ILDs pathogenesis and progression (39). In total, 50 clinically significant SNPs were reported in 9 genes potentially associated with IPF (Supplementary Table S3). Out of them, 29, 6, 5 and 3 SNPs were associated with VWF, NOD2, TERT and ABCA3 genes, respectively (Supplementary Table S3). Mutations in surfactant-production-and-function genes (SP-B, SP-C and ABCA3), surfactant catabolism (GM-CSF receptor) and transcription factors involved in lung development (FOXF1) are likely contributing factors to many forms of ILDs including ChILDs (40). Mutations in the telomerase genes (TERT and TERC) were also detected very frequently in ILDs patients (41).

#### SNP, miRNA and DCGs association

Polymorphisms in miRNAs (miR-polymorphisms) are emerging as a comparatively new and effective tool to be used in disease biology, diagnosis and prognosis (42). Ongoing cohort studies proved that miR-polymorphisms can regulate a variety of biological and pathological processes such as cell growth, differentiation, apoptosis and tumorigenesis (43). A unique association analysis among SNP, miRNA and DCGs (miR-polymorphisms) was performed and incorporated in ILDgenDB. In total, 9170 miR-polymorphisms associations were identified for 91 DCGs. All the associations can directly be accessed using ‘miRNA-SNP correlation’ option under ‘Disease’ or ‘miRNAs AND/OR SNPs’ as query option of the ILDgenDB resource. SNPs in miRNAs can cause an alteration in existing binding sites to produce illicit binding sites. The altered binding of miRNA-mRNA duplex may cause aberrant gene expression which can potentially contribute to disease susceptibility (44). Other than ILDs, the potential roles of SNP rs17281995 in miR-337, miR-582, miR-200a, miR-184 and miR-212 (45), and rs11614913 in hsa-mir-196a2 are also reported in cancer pathogenesis and diagnostics (46). Out of 9170 miR-polymorphisms associations, 20 different genes: HMGA2, SPRED1, PLCG1, FKBP1A, FAM13A, DLG1, TSC1, NF1, FBN1, PIK3C2A, BDNF, FOXF1, MDGA2, CTGF, CXCL12, IL10, CAV1, NOG, FASLG and ITGA3 were found to have highest number of association. These 20 DCGs were associated with 5706 miR-polymorphisms indicated that these are from 229 different miRNAs, and the numbers of distinct SNPs in these miRNAs are 340 (Supplementary Table S4). These outcomes suggest that these miRNAs may further be experimentally validated to determine their potential role in ILD or its subtypes diagnosis and prognosis.

### Biomarkers and their characterization

Biomarkers play crucial roles in the disease diagnostics, prognostics and the development of molecular target-based therapeutics (47). Integrated disease-specific genetic data resources can provide a better opportunity for identifying new biomarkers to develop novel diagnosis strategies (48). In order to provide integrated information, ILDs biomarkers were collected from published literature. Their annotations were manually verified and incorporated into ‘Biomarker’ section of ILDgenDB resource (Figure 3D). In total, 216 biomarkers were categorized as disease-specific protein (5), disease-specific (135), protein involved in pathogenic pathway (57), serum (40), disease-specific serum (7), protein (10), disease-specific miRNA (3) and gene (12). Majority of these biomarkers are used to determine whether the patient has ILD disease or not. However, very few biomarkers including KL6, SP-A, SP-D etc. are available to determine subtypes of ILDs. To provide a better understanding, ‘Source’ (related literature reference) and remarks related to these biomarkers are also provided in ILDgenDB.

Identification of DCGs mutations or altered expression which can disturb different molecular pathways and cellular processes have potential to be used as a biomarker for disease diagnosis and/or prognostication (49, 50). SNPs, differential expression of miRNAs, miR-polymorphism, different pathways and other molecular function of these 299 DCGs were analyzed. New biomarkers are proposed on the basis of above analysis. DCGs mapped with significant GO and maximum number of pathways (top 10 interactions) are referred as metabolic biomarkers. SNPs and miRNAs with a high degree of correlation with DCGs, and SNPs associated with miRNAs regions are represented as bio-molecular biomarkers (Supplementary Table S5). All these potential biomarkers require further validation to determine their diagnostic potential (Figure 2).

### Discussion and future developments

To date, several databases were reported on the basis of gene-disease mapping or associations of genetic factors with disease. For example, ClinVar provides gene-associated variations and phenotypes with their clinical significance. Similarly, SNPs-3D (51) provides SNPs. T-HOD (hypertension, obesity and diabetes) (52) and PubMath (cancer methylation) (53) are text-mining technologies associated with disease-specific databases for disease-gene mapping. Integrated database ‘DISEASES’ contains manually curated data related to disease-gene associations, cancer mutation and genome-wide association (17). Similarly DisGeNET provides gene, disease and SNPs information, but information about miRNAs or biomarkers have not been provided (51). However, detailed information about ILDs association with genes and genetic data is limited. To date, ILDgenDB is the only integrated database providing information on manually curated DCGs and their associations with miRNAs, SNPs, GO, pathways and biomarkers. Additionally, ILDgenDB also provides miRNAs, SNPs, miR-Polymorphism data, and the functional analyses such as GO and pathways association for DCGs. Characterization of potential biomarkers that have a strong correlation with DCGs was performed to validate and enhance the utility of this resource. Another case-study was also performed using a subset of ncRNAs and pathways from ILDgenDB (54). This study signified the role of ncRNAs (hsa-miR-1, hsa-miR-335-5p etc.) in ILDs-associated regulatory pathways (Cytokine-cytokine receptor interaction, TGF Beta Signaling Pathway etc.). Similarly, hsa-mir-575, hsa-mir-665, hsa-mir-4417 etc. found to be up-regulated in ILDs expression data when compared to healthy controls (54). These ncRNAs biomarkers may facilitate genetic-based disease monitoring and diagnosis. This resource is expected to serve as a highly useful unique repository to get an overview of biology of the genes involved in ILDs. Majority of published DCGs are included in the current release, but our foremost objective is to integrate more DCGs to this resource. Also, in the current version, limited significant references for a given DCGs were covered. Therefore, in future updates, authors plan to add updated references for DCGs in the current version to maintain quality and utility of the resource. ILDgenDB will be updated periodically to make the resource up-to-date with new findings of ILDs.

## Conclusions

ILDgenDB is a centralized repertoire that hosts a wide range of ILDs data related to genes, proteins, biomarkers etc. and their analyses. This knowledge resource aims to provide a comprehensive platform for different utilities such as unrestricted public access of annotated datasets, and to enhance the efficacy of disease management and monitoring through contemporary genetic data. This resource contains 299 literature curated DCGs, and their associations with SNPs, miRNA, pathways, biological processes etc. The DCGs involvement in ILDs was verified by mining disease-related data from databases such as GAD, OMIM, GHR, CTD, DISEASE and GeneCards. The outcomes of these DCGs, GO, pathway and phenotypic analyses have indicated the potential role of the immune systems, molecular binding and inflammatory host defenses, and selective immune processes pathways in the pathogenesis of disease. Association studies implicated many miRNAs and SNPs for the expression alteration of DCGs. Association analysis among SNP-miRNA-DCGs (miR-polymorphisms) was performed. A total of 229 miRNAs with 340 distinct SNPs were associated with 20 different ILDs genes. These miR-polymorphisms were proved to have potential role in cancers and other disease, and similar could be done to determine and validate their potential role in ILDs or its subtypes diagnosis and prognosis. Some important identified DCGs such as cytokines, interleukins, surfactant proteins etc. may play a vital role in disease pathogenesis. Experimental validations of novel DCGs, miRNA etc. can be valuable assets for the identification of new biomarkers. Datasets and their analyses available in ILDgenDB would be helpful to researchers from diverse backgrounds working for the betterment of ILDs. This resource will be periodically updated with enhanced features and information. ILDgenDB will assist in improving existing knowledge about ILDs.

## Supplementary data

Supplementary data are available at *Database* Online.

## Supplementary Material

Supplementary Data
