# Supplementary material for: ILDgenDB: integrated genetic knowledge resource for interstitial lung diseases (ILDs)
Source: Database (Oxford). 2018 Jun 9;2018:bay053. doi: 10.1093/database/bay053 (PMC6007225; doi:10.1093/database/bay053)
Supplement: Supplementary Data [file bay053_supp.docx]

**Supplementary Material**

**ILDgenDB: Integrated genetic knowledge resource for interstitial lung diseases (ILDs)**

Smriti Mishra^1*^, Mohammad I Shah^1^, Malay Sarkar^2^, Nimisha Asati^1^, Chittaranjan Rout^1^

^1^Department of Biotechnology & Bioinformatics, Jaypee University of Information Technology, Waknaghat, Solan, Himachal Pradesh, 173234, India

^2^Department of Pulmonary Medicine, Indira Gandhi Medical College, Shimla, 171001, India

*Email: [smriti.mishra1906@gmail.com](mailto:smriti.mishra1906@gmail.com)

Gene Ontology (GO) mapping of all the DCGs in three different GO domains: Molecular function (A), Cellular component (B) and Biological process (C)


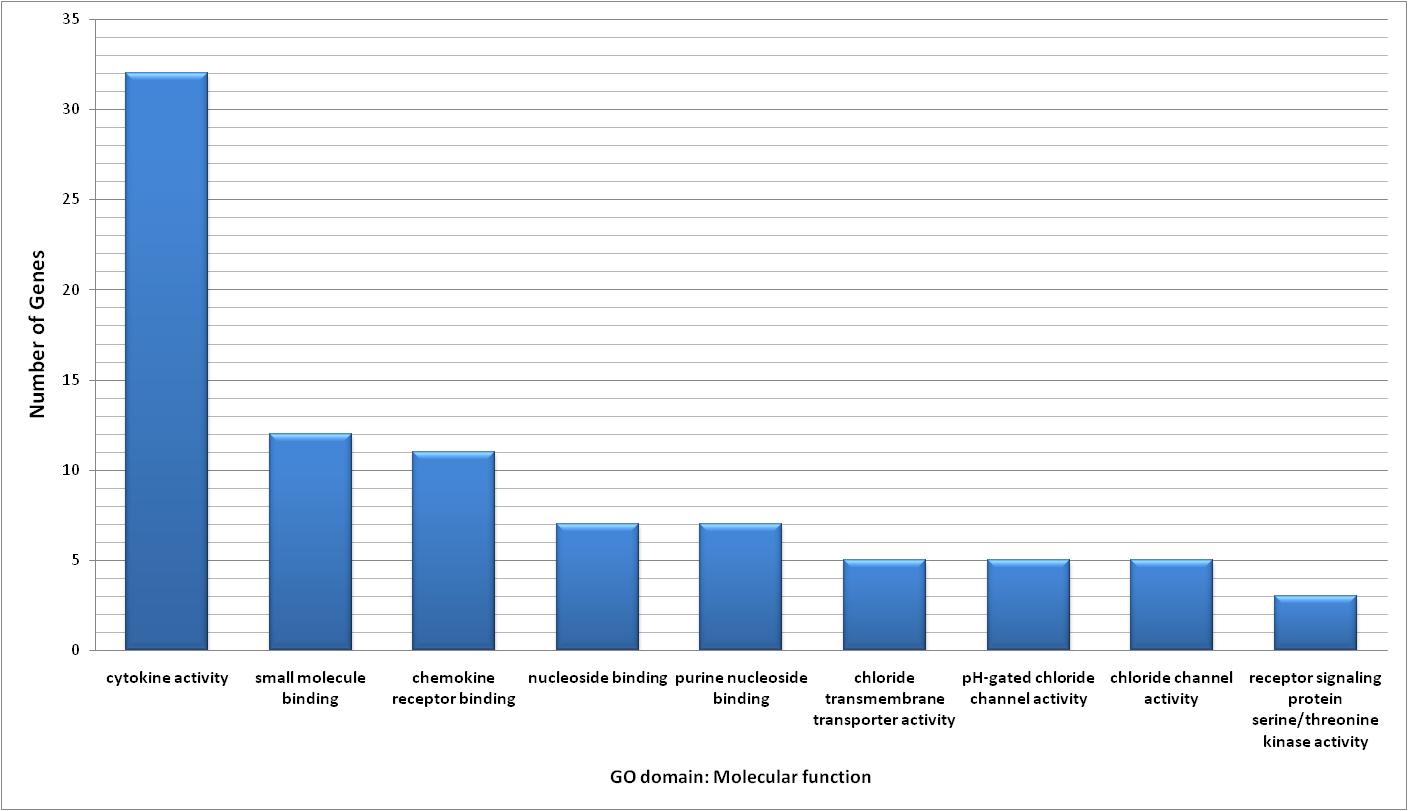


Fig. S1, A: Distribution of DCGs with different molecular function


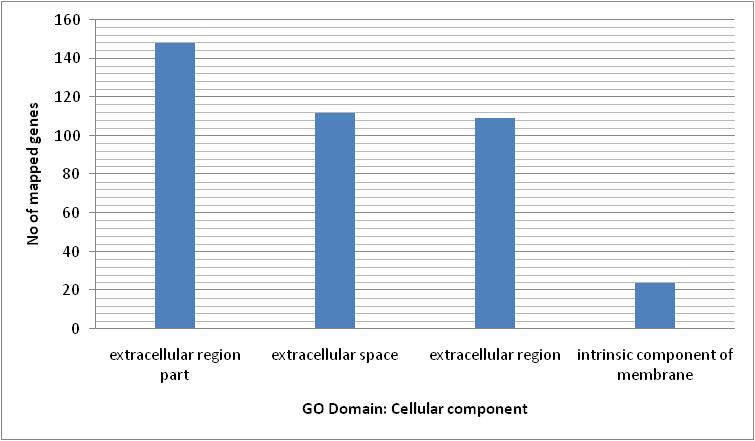


Fig. S1, B: Distribution of DCGs with different cellular component


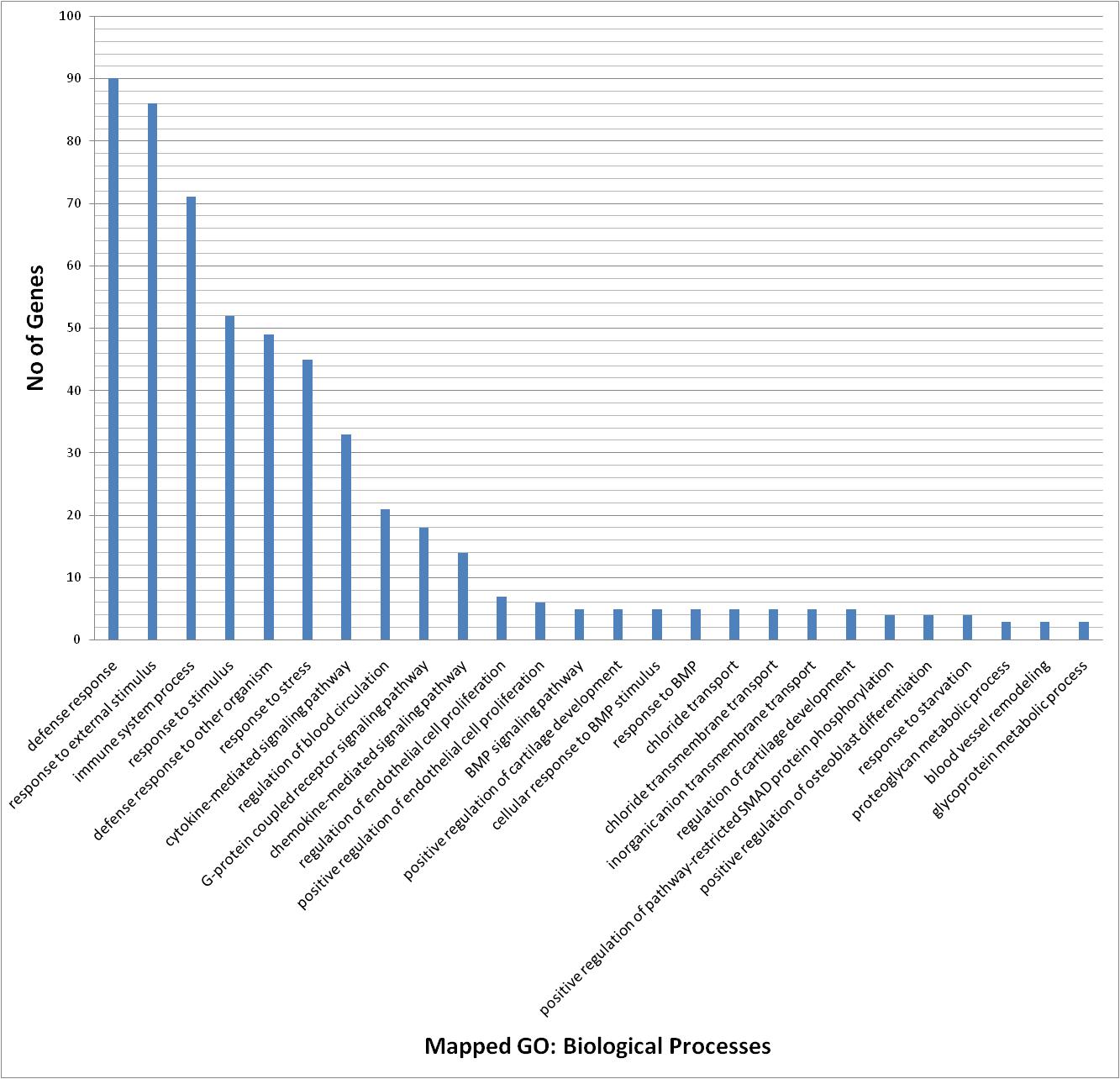


Fig. S1, C: Distribution of DCGs with different biological processes

Table S1: Web resources and databases used or referred to establish ILDgenDB knowledge resources

| S. No | Data resource^*^ | Web link |
| --- | --- | --- |
| 1 | Amigo | http://amigo.geneontology.org/amigo |
| 2 | DAVID | https://david.ncifcrf.gov/ |
| 3 | DbSNP | https://www.ncbi.nlm.nih.gov/SNP/ |
| 4 | Disease | http://www.diseasesdatabase.com/ |
| 5 | Ensembl | http://asia.ensembl.org/index.html |
| 6 | Ensembl Biomart | www.ensembl.org/biomart |
| 7 | Entrez | https://www.ncbi.nlm.nih.gov/gquery/ |
| 8 | GAD | https://geneticassociationdb.nih.gov/ |
| 9 | Genebank | https://www.ncbi.nlm.nih.gov/genbank/ |
| 10 | Genecard | http://www.genecards.org/ |
| 11 | GHR | https://ghr.nlm.nih.gov/ |
| 12 | HGNC | http://www.genenames.org/ |
| 13 | Interpro | https://www.ebi.ac.uk/interpro/ |
| 14 | KEGG | http://www.genome.jp/kegg/ |
| 15 | MESH | https://www.ncbi.nlm.nih.gov/mesh |
| 16 | MGI | http://www.informatics.jax.org/ |
| 17 | MIRBASE | http://www.mirbase.org/ |
| 18 | Mirdsnp | http://mirdsnp.ccr.buffalo.edu/ |
| 19 | MIRWALK | http://zmf.umm.uni-heidelberg.de/apps/zmf/mirwalk2/index.html |
| 20 | OMIM | https://www.omim.org/ |
| 21 | Panther | http://www.pantherdb.org/ |
| 22 | PDB | http://www.rcsb.org/pdb/home/home.do |
| 23 | PITA | https://omictools.com/pita-tool |
| 24 | Polyphen-2 | http://genetics.bwh.harvard.edu/pph2/ |
| 25 | Pubmed | https://www.ncbi.nlm.nih.gov/pubmed |
| 26 | SIFT | http://sift.jcvi.org/ |
| 27 | Starbase | http://starbase.sysu.edu.cn/ |
| 28 | Targetscan | http://www.targetscan.org/vert_71/ |
| 29 | UCSC | https://genome.ucsc.edu/ |
| 30 | Uniprot | http://www.uniprot.org/ |
| 31 | Vega | <http://vega.sanger.ac.uk/index.html> |

^*^ All the web resources were used after agreement of their terms and conditions. Data retrieved from web resources were used after manual analysis and filtration.

Table S2: Top 10 potential Pathways mapped to disease candidate genes (DCGs)

| **S. No** | **Pathway ID** | **Pathway Name** | **Number of DCGs mapped** |
| --- | --- | --- | --- |
| 1 | P00031 | Inflammation mediated by chemokine and cytokine signaling pathway | 26 |
| 2 | P00036 | Interleukin signaling pathway | 18 |
| 3 | P06664 | Gonadotropin-releasing hormone receptor pathway | 13 |
| 4 | P00052 | TGF-beta signaling pathway | 9 |
| 5 | P00054 | Toll receptor signaling pathway | 8 |
| 6 | P00011 | Blood coagulation | 8 |
| 7 | P00006 | Apoptosis signaling pathway | 4 |
| 8 | P00005 | Angiogenesis | 4 |
| 9 | P00034 | Integrin signalling pathway | 4 |
| 10 | P00057 | Wnt signaling pathway | 4 |

Table S3: Manually curated SNPs and associated DCGs potentially involved in IPF pathogenesis

| **S.No.** | **DCGs** | **Count of SNPs** |
| --- | --- | --- |
| 1 | VWF | 29 |
| 2 | NOD2 | 6 |
| 3 | TERT | 5 |
| 4 | ABCA3 | 3 |
| 5 | PRF1 | 2 |
| 6 | TNC | 2 |
| 7 | IL2RA | 1 |
| 8 | TGFB1 | 1 |
| 9 | VDR | 1 |
| Grand Total | | 50 |

Top targeted DCGs, and SNPs frequency in those DCGs involved in IPF. Analysis suggested that maximum number of SNPs have been predicted in VWF (29).

Table S4: Co-occurrence of miRNAs-SNPs in DCGs

| S. No. | DCGs | Count of SNPs | Count of miRNAs |
| --- | --- | --- | --- |
| 1 | HMGA2 | 34 | 11 |
| 2 | FGF2 | 19 | 1 |
| 3 | VDR | 18 | 1 |
| 4 | CTGF | 17 | 5 |
| 5 | CAV1 | 13 | 1 |
| 6 | STAT3 | 12 | 2 |
| 7 | CXCL12 | 11 | 1 |
| 8 | BDNF | 9 | 3 |
| 9 | HOXA5 | 9 | 1 |
| 10 | FBN1 | 8 | 1 |
| 11 | IL6 | 5 | 1 |
| 12 | ELMOD2 | 4 | 1 |

Table S5: Proposed candidate biomarkers on the basis of functional and structural mapping with DCGs

| **Candidate biomarkers** | **Number of interactions** | **Remarks** |
| --- | --- | --- |
| DNAL1 | 175 | DCGs targeted by muliple number of miRNAs (Interaction= DCGs-miRNA interactions) |
| MICA | 143 |  |
| FGF2 | 99 |  |
| C3 | 94 |  |
| ITGA3 | 79 |  |
| ADM | 77 |  |
| SERPINH1 | 77 |  |
| PTGIS | 74 |  |
| HMGA2 | 73 |  |
| CCL22 | 71 |  |
| SLC6A4 | 66 |  |
| STAT3 | 63 |  |
| CAV1 | 58 |  |
| ABCF2 | 56 |  |
| CPS1 | 56 |  |
| EDN1 | 56 |  |
| AQP3 | 53 |  |
| GDNF | 51 |  |
| IL2RA | 51 |  |
| KCNK3 | 51 |  |
| BLOC1S3 | 49 |  |
| BLOC1S6 | 49 |  |
| BMPR2 | 49 |  |
| LACTB | 47 |  |
| CD209 | 46 |  |
| HMOX1 | 46 |  |
| hsa-miR-335 | 65 | miRNAs having multiple number of DCGS target (Interaction= DCGs-miRNA interactions) |
| hsa-miR-26b | 34 |  |
| hsa-let-7 | 33 |  |
| hsa-miR-30 | 26 |  |
| hsa-miR-20 | 24 |  |
| hsa-miR-124 | 24 |  |
| hsa-miR-17 | 17 |  |
| hsa-miR-93 | 17 |  |
| hsa-miR-106b | 16 |  |
| hsa-miR-4722 | 15 |  |
| hsa-miR-1 | 14 |  |
| hsa-miR-155 | 14 |  |
| hsa-miR-16 | 14 |  |
| hsa-miR-6778 | 14 |  |
| hsa-mir-8485 | 14 |  |
| hsa-miR-98 | 14 |  |
| hsa-miR-128 | 13 |  |
| hsa-miR-19a | 13 |  |
| hsa-miR-21 | 13 |  |
| hsa-miR-3653 | 13 |  |
| hsa-miR-519d | 13 |  |
| hsa-miR-4768 | 12 |  |
| hsa-miR-92a | 12 |  |
| hsa-miR-106a | 11 |  |
| hsa-miR-125b | 11 |  |
| hsa-miR-149 | 11 |  |
| hsa-miR-186 | 11 |  |
| hsa-miR-192 | 11 |  |
| hsa-miR-193b | 11 |  |
| hsa-miR-548c | 11 |  |
| Inflammation mediated by chemokine and cytokine signaling pathway (P00031) | 26 | Potential Pathways mapped maximum number of disease candidate genes (DCGs) ((Interaction= DCGs mapped with pathways) |
| Interleukin signaling pathway (P00036) | 18 |  |
| Gonadotropin-releasing hormone receptor pathway (P06664) | 13 |  |
| TGF-beta signaling pathway (P00052) | 9 |  |
| Toll receptor signaling pathway (P00054) | 8 |  |
| Blood coagulation (P00011) | 8 |  |
| Apoptosis signaling pathway (P00006) | 4 |  |
| Angiogenesis (P00005) | 4 |  |
| Integrin signalling pathway (P00034) | 4 |  |
| Wnt signaling pathway (P00057) | 4 |  |
| HMGA2 | 1183 | Top targeted DCGs mapped with maximum number of clinically confirmed mir-polymorphism ((Interaction= DCGs-miRNA-SNP interactions) |
| SPRED1 | 496 |  |
| PLCG1 | 450 |  |
| FKBP1A | 357 |  |
| FAM13A | 304 |  |
| DLG1 | 300 |  |
| TSC1 | 257 |  |
| NF1 | 251 |  |
| FBN1 | 220 |  |
| PIK3C2A | 213 |  |
| BDNF | 206 |  |
| FOXF1 | 203 |  |
| MDGA2 | 196 |  |
| CTGF | 194 |  |
| CXCL12 | 194 |  |
| IL10 | 191 |  |
| CAV1 | 174 |  |
| NOG | 161 |  |
| FASLG | 156 |  |
| ITGA3 | 153 |  |
| Defense Response (Go:0006952) | 90 | Top Gene Ontology (Biological process) terms mapped with maximum number of DCGs (Interaction= Number of DCGs with mapped GO term) |
| Response To External Stimulus(Go:0009605) | 86 |  |
| Immune System Process (Go:0002376) | 71 |  |
| Response To Stimulus (Go:0050896) | 52 |  |
| Defense Response To Other Organism (Go:0098542) | 49 |  |
| Response To Stress(Go:0006950) | 45 |  |
| Cytokine-Mediated Signaling Pathway(Go:0019221) | 33 |  |
| Regulation Of Blood Circulation(Go:1903522) | 21 |  |
| G-Protein Coupled Receptor Signaling Pathway(Go:0007186) | 18 |  |
| Chemokine-Mediated Signaling Pathway(Go:0070098) | 14 |  |
| Cytokine Activity(Go:0005125) | 32 | Top Gene Ontology (Moleculer function) terms mapped with maximum number of DCGs (Interaction= Number of DCGs with mapped GO term) |
| Small Molecule Binding(Go:0036094) | 12 |  |
| Chemokine Receptor Binding(Go:0042379) | 11 |  |
| Nucleoside Binding(Go:0001882) | 7 |  |
| Purine Nucleoside Binding(Go:0001883) | 7 |  |
| Chloride Transmembrane Transporter Activity(Go:0015108) | 5 |  |
| Ph-Gated Chloride Channel Activity(Go:0061797) | 5 |  |
| Chloride Channel Activity(Go:0005254) | 5 |  |
| Receptor Signaling Protein Serine/ Threonine Kinase Activity (Go:0004702) | 3 |  |
| Extracellular Region Part(Go:0044421) | 148 | Top Gene Ontology (Cellular component) terms mapped with maximum number of DCGs (Interaction= Number of DCGs with mapped GO term) |
| Extracellular Space (Go:0005615) | 112 |  |
| Extracellular Region (Go:0005576) | 109 |  |
| Intrinsic Component Of Membrane (Go:0031224) | 24 |  |
